# Supplementary material for: Secretome translation shaped by lysosomes and lunapark-marked ER junctions
Source: Nature. 2025 Nov 5;649(8095):227–36. doi: 10.1038/s41586-025-09718-0 (PMC12727531; doi:10.1038/s41586-025-09718-0)
Supplement: Supplementary file 1 — This file contains uncropped western blot images corresponding to Fig. 5e and Extended Data Fig. 5c,d, Supplementary Figs. 1 and 2 and Supplementary Tables 1–3. [file 41586_2025_9718_MOESM1_ESM.pdf]

nature portfolio

https://doi.org/10.1038/s41586-025-09718-0

Supplementary information

Secretome translation shaped by lysosomes and lunapark-marked ER junctions

In the format provided by the authors and unedited

Supplementary Information

I. Uncropped images of western blot for Fig. 5e
II. Uncropped images of western blot for Extended Data Fig. 5c
III. Uncropped images of western blot for Extended Data Fig. 5d
IV. Motion-based analysis and its assumptions
  a. Supplementary Fig. 1
  b. Supplementary Fig. 2
V. Supplementary Tables
  a. Supplementary Table 1**.** List of Plasmids for this study
  b. Supplementary Table 2. siRNA and qPCR primers used for this study
  c. Supplementary Table 3. Single guide RNAs for generating Lunapark KO U-2 OS cells
VI. Supplementary Videos
  a. Supplementary Video 1
  b. Supplementary Video 2
  c. Supplementary Video 3
  d. Supplementary Video 4
  e. Supplementary Video 5
  f. Supplementary Video 6
  g. Supplementary Video 7
  h. Supplementary Video 8
  i. Supplementary Video 9

I. Uncropped images of western blot for Fig. 5e

anti-tubulin for p-eIF2S1

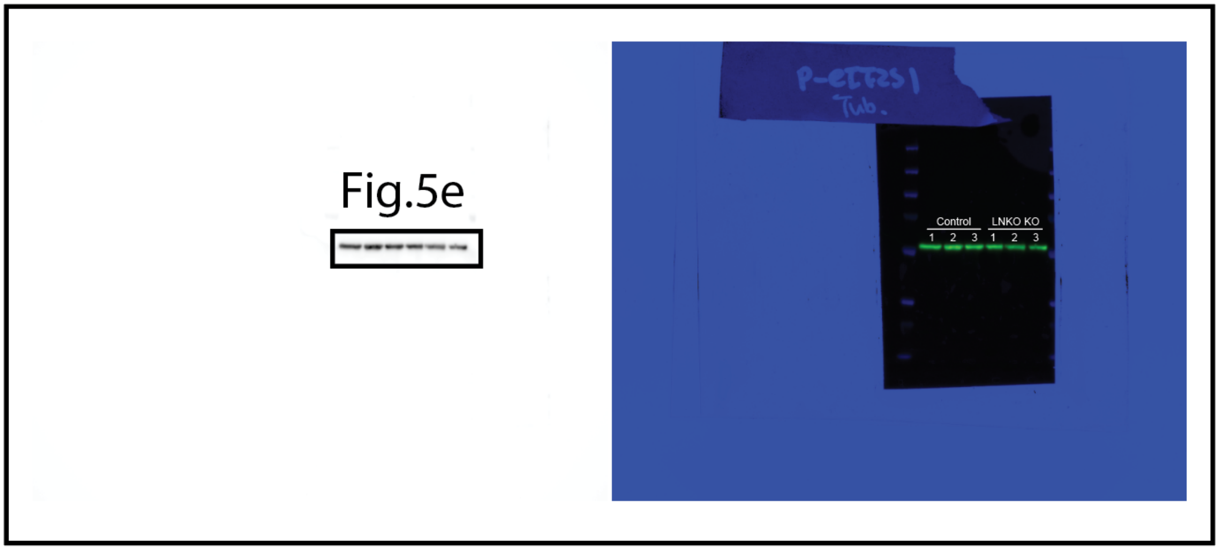

anti-p-eIF2S1

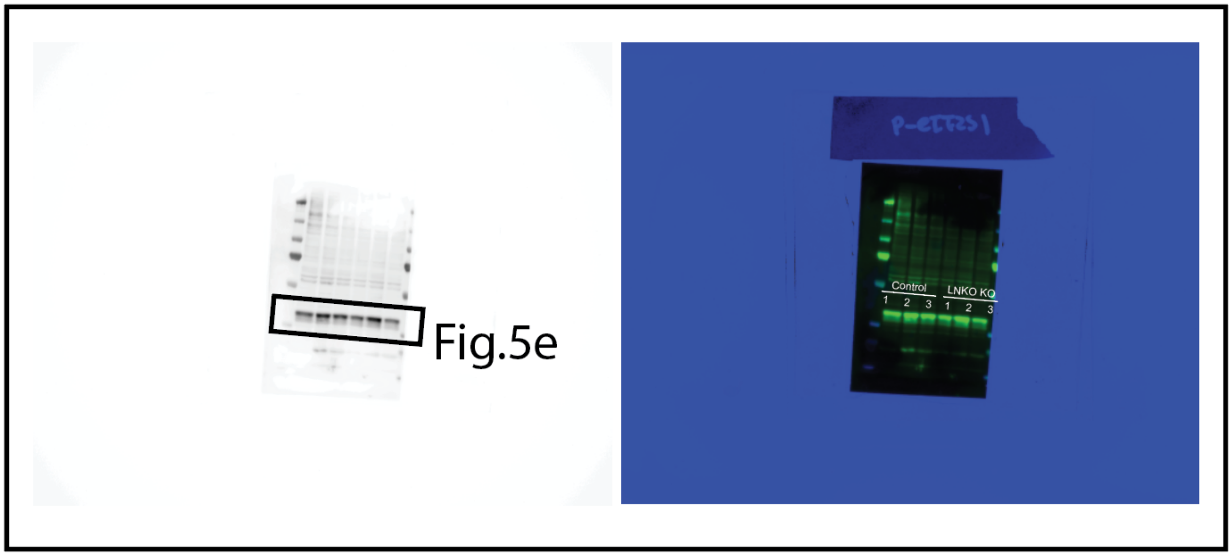

anti-tubulin for eIF2S1

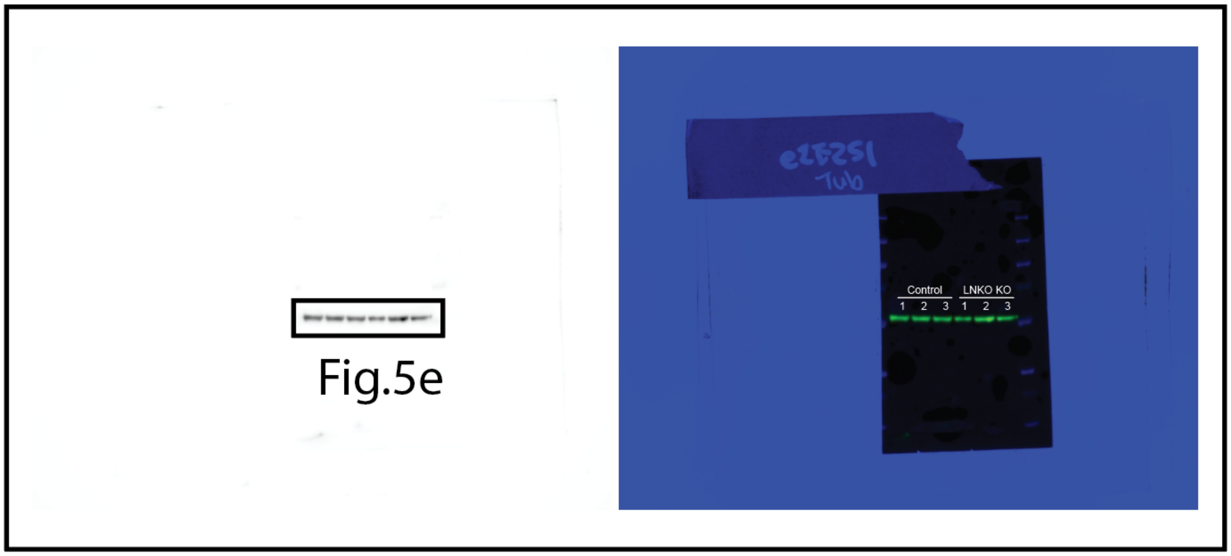

anti-eIF2S1

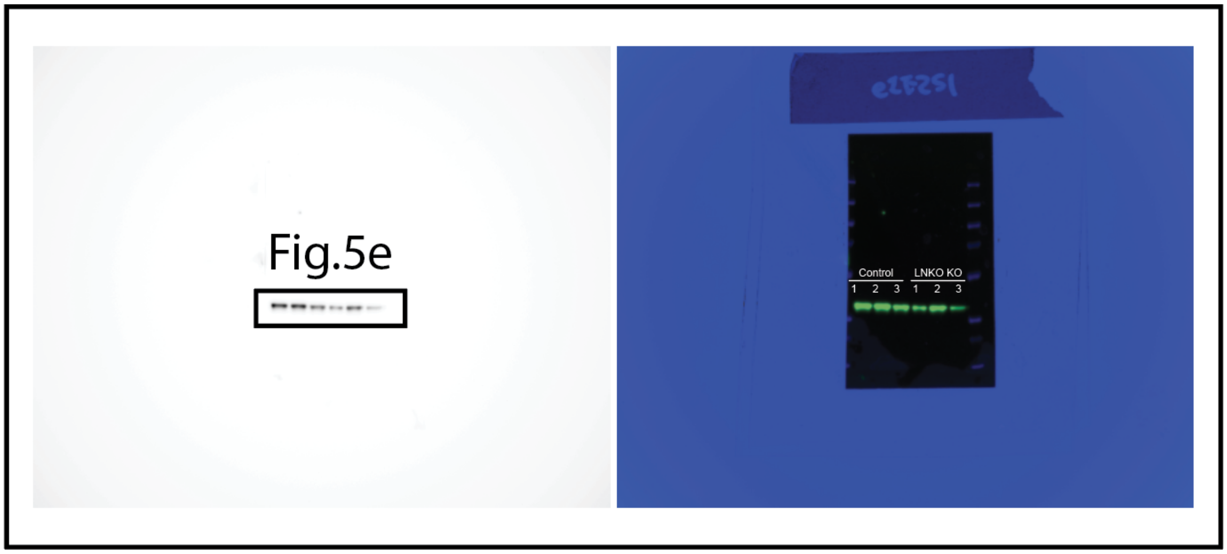

anti-tubulin for LNPK

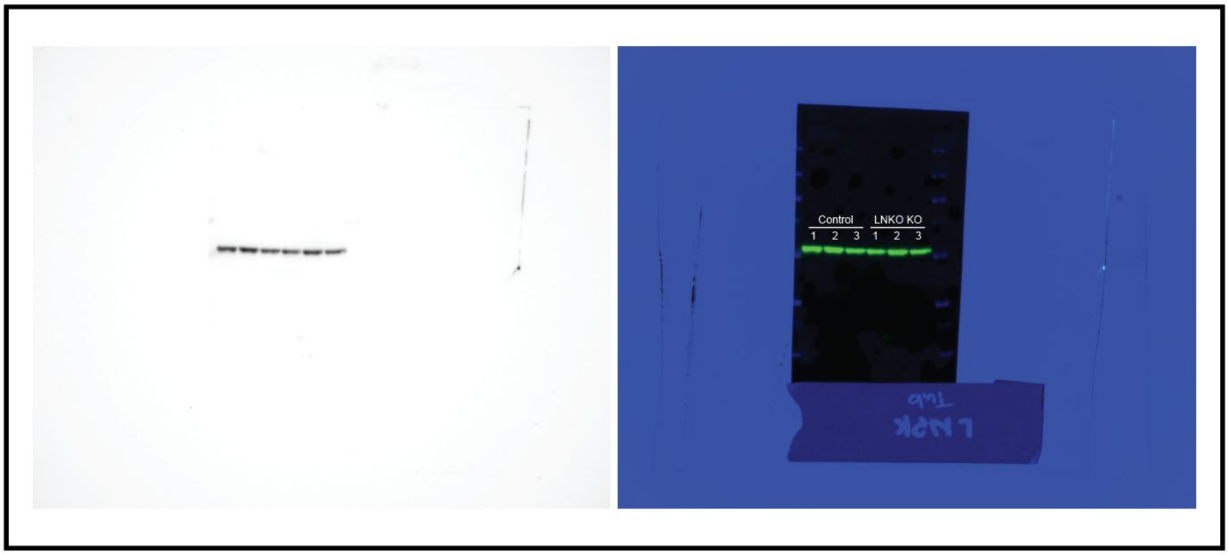

anti-LNPK

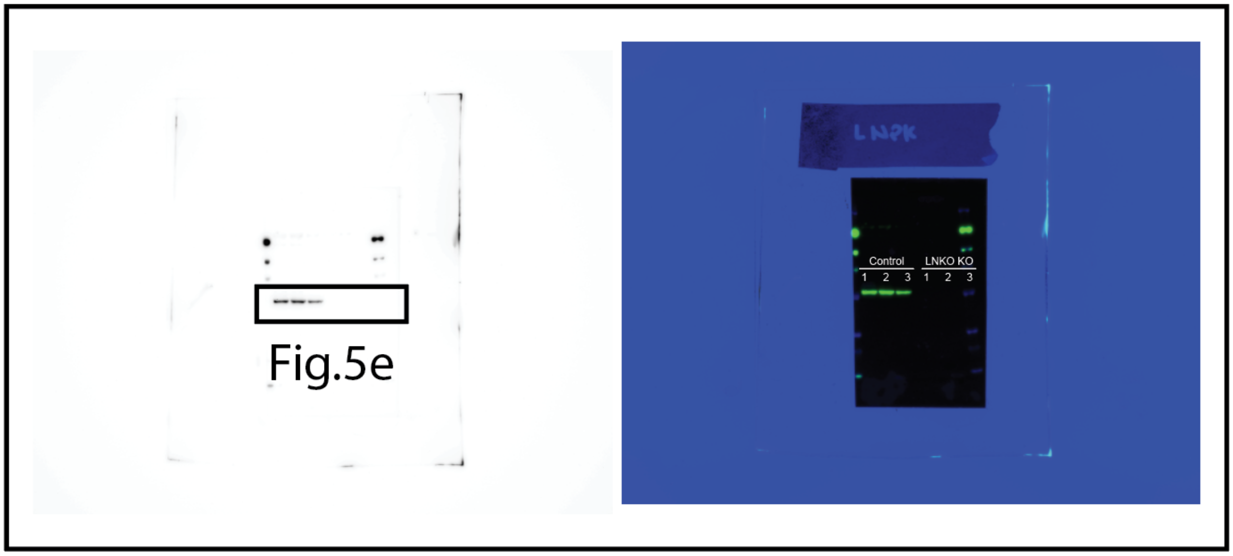

II. Uncropped images of western blot for Extended Data Fig. 5c

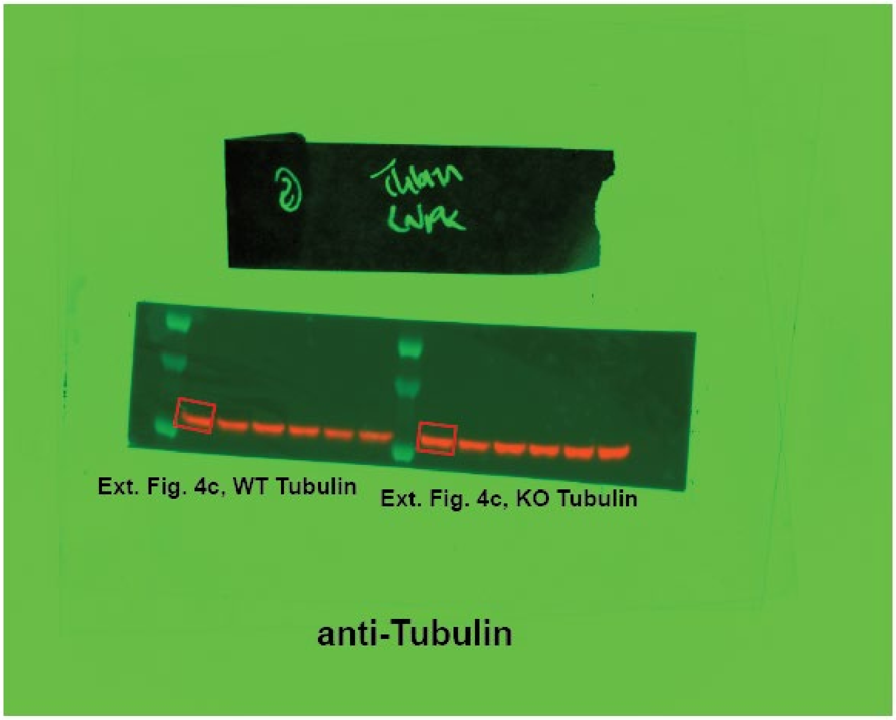

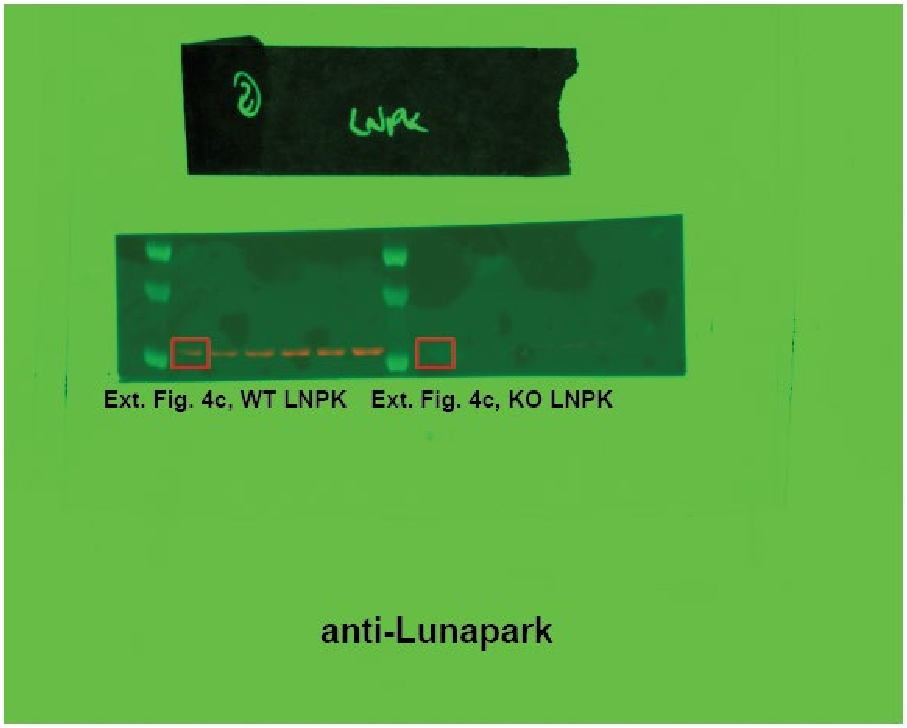

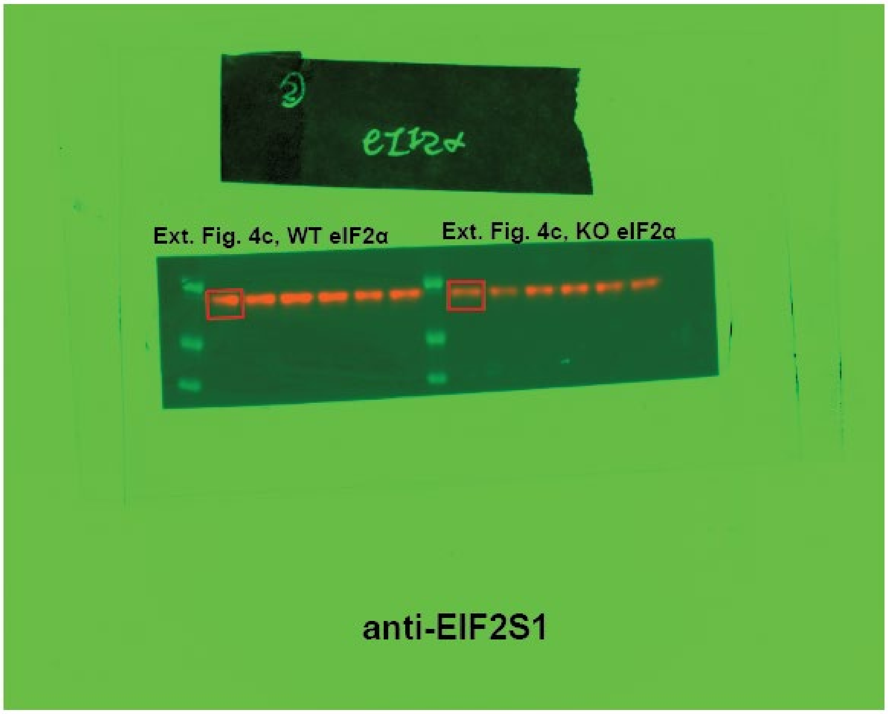
Ext. Fig. 4c, WT eIF2α
Ext. Fig. 4c, KO eIF2α
anti-EIF2S1

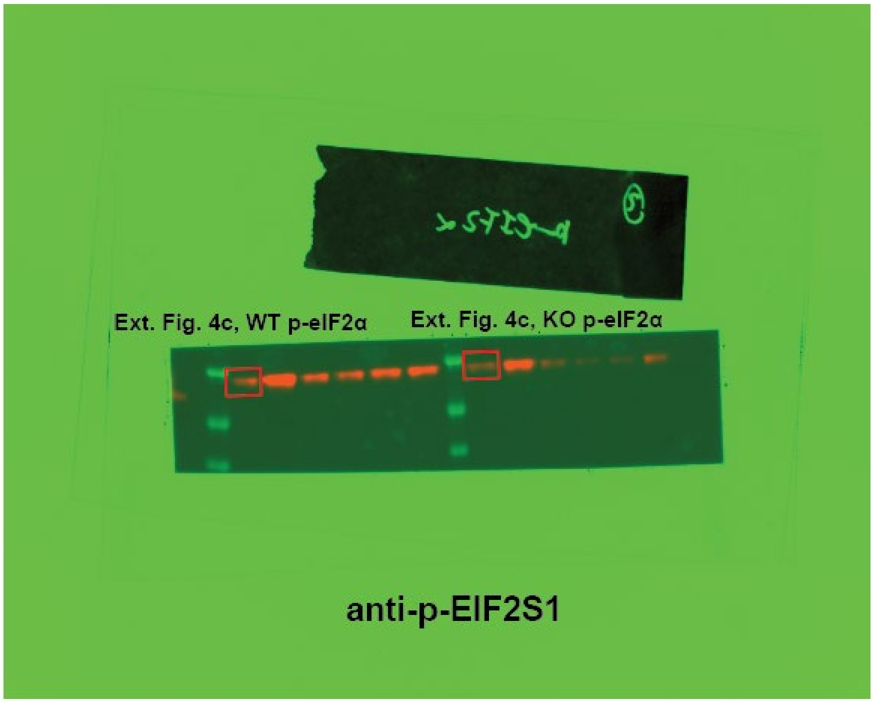
Ext. Fig. 4c, WT p-eIF2α
Ext. Fig. 4c, KO p-eIF2α
anti-p-EIF2S1

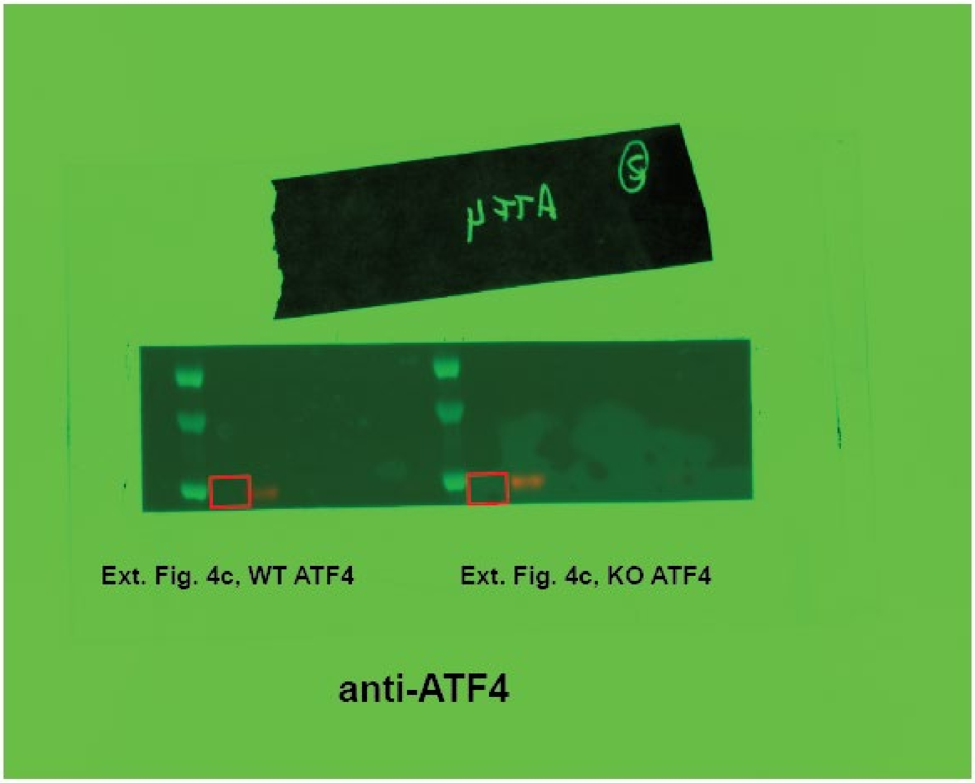
Ext. Fig. 4c, WT ATF4
Ext. Fig. 4c, KO ATF4
anti-ATF4

III. Uncropped images of western blot for Extended Data Fig. 5d

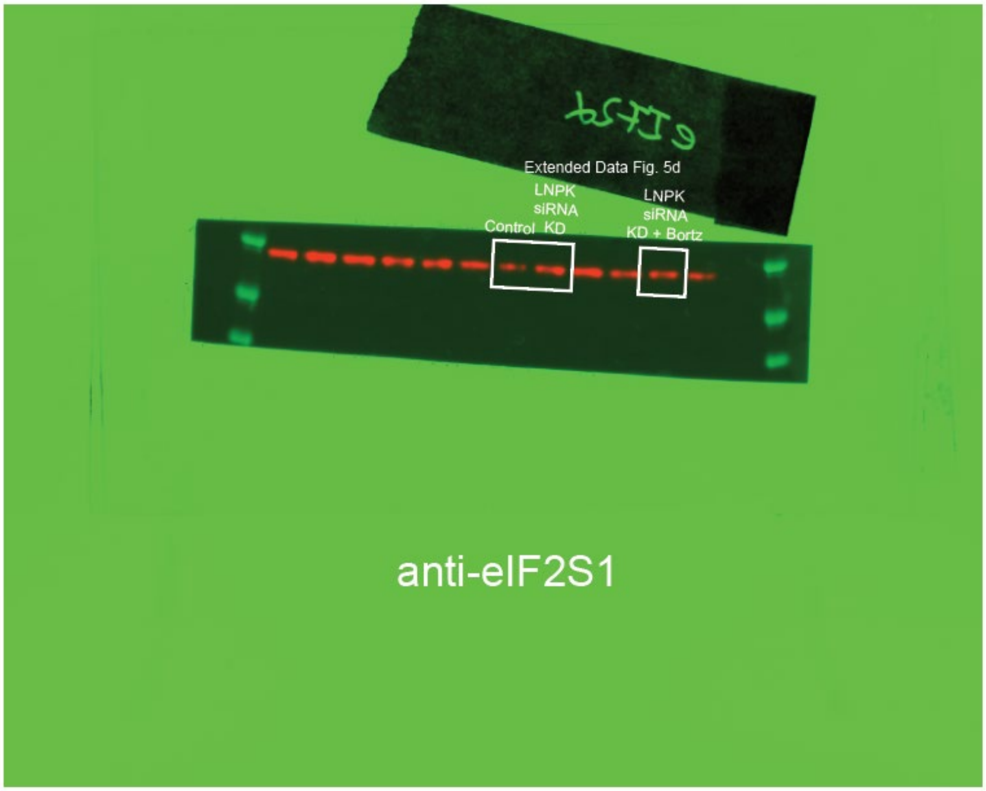

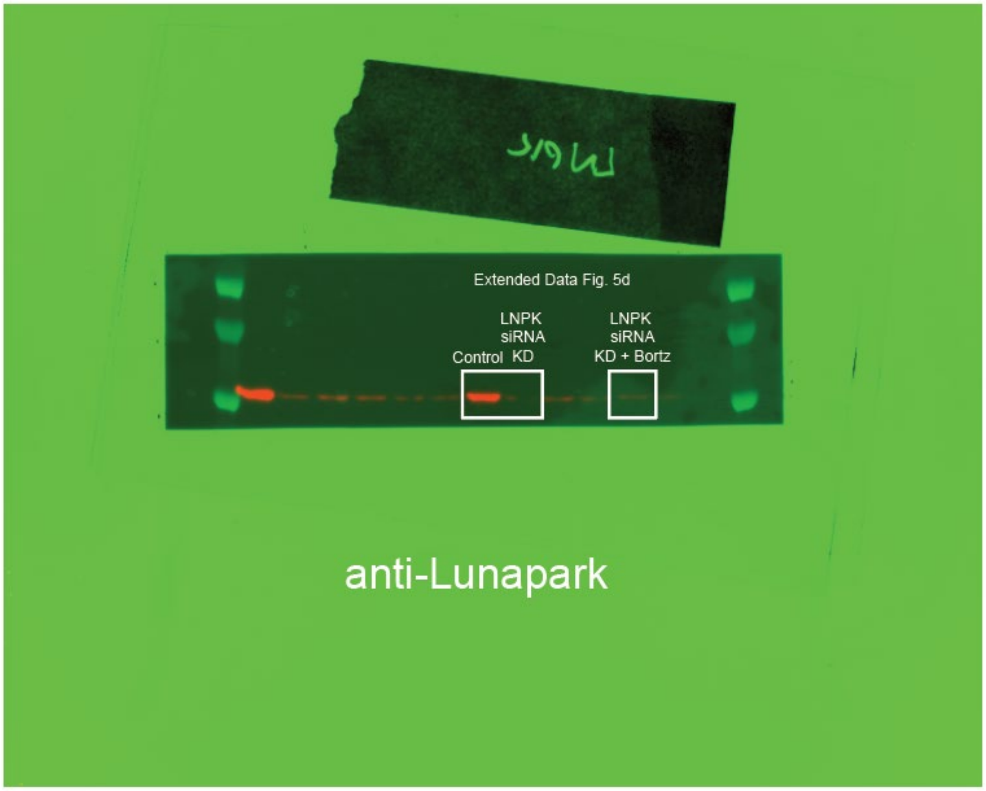
Extended Data Fig. 5d
Control
LNPK siRNA KD
LNPK siRNA KD + Bortz
anti-Lunapark

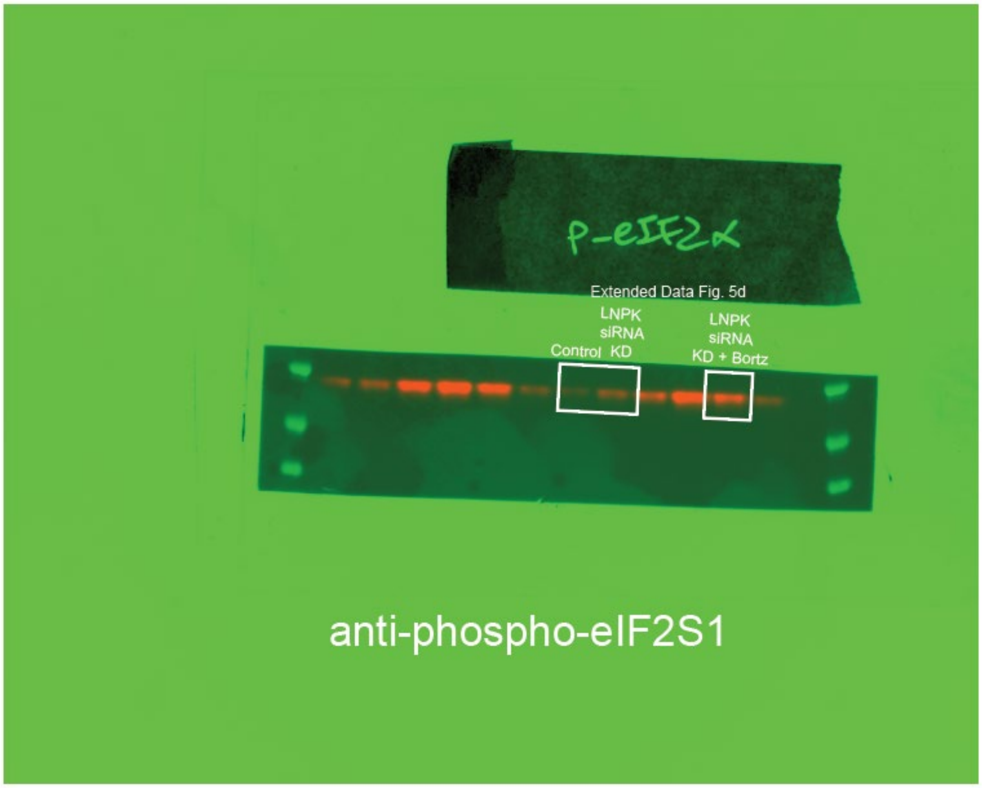
p-eIF2α
Extended Data Fig. 5d
Control
LNPK siRNA KD
LNPK siRNA KD + Bortz
anti-phospho-eIF2S1

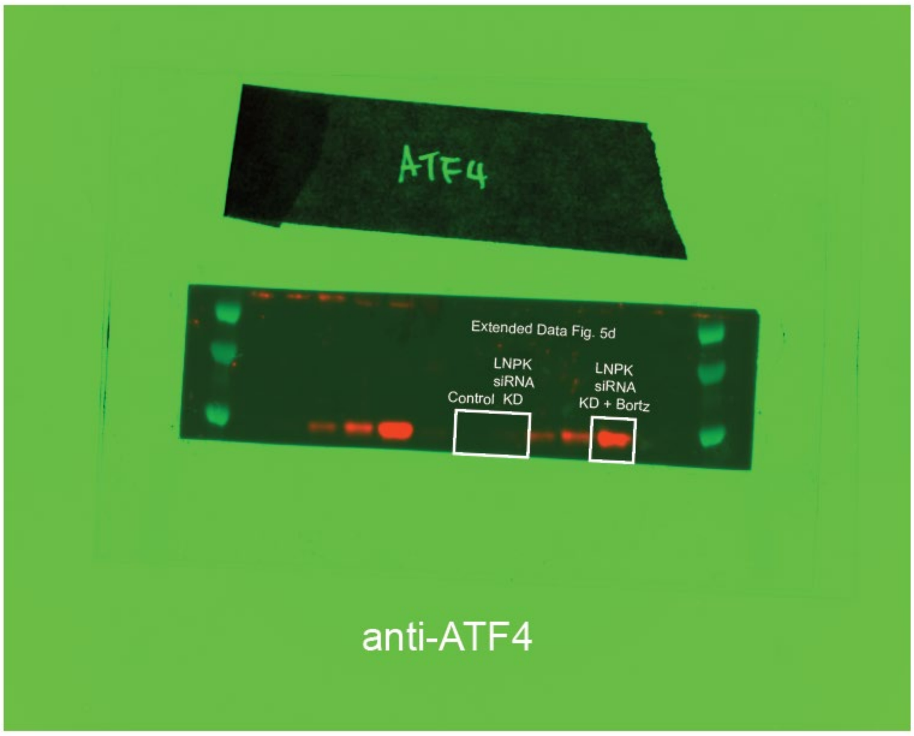

anti-ATF4

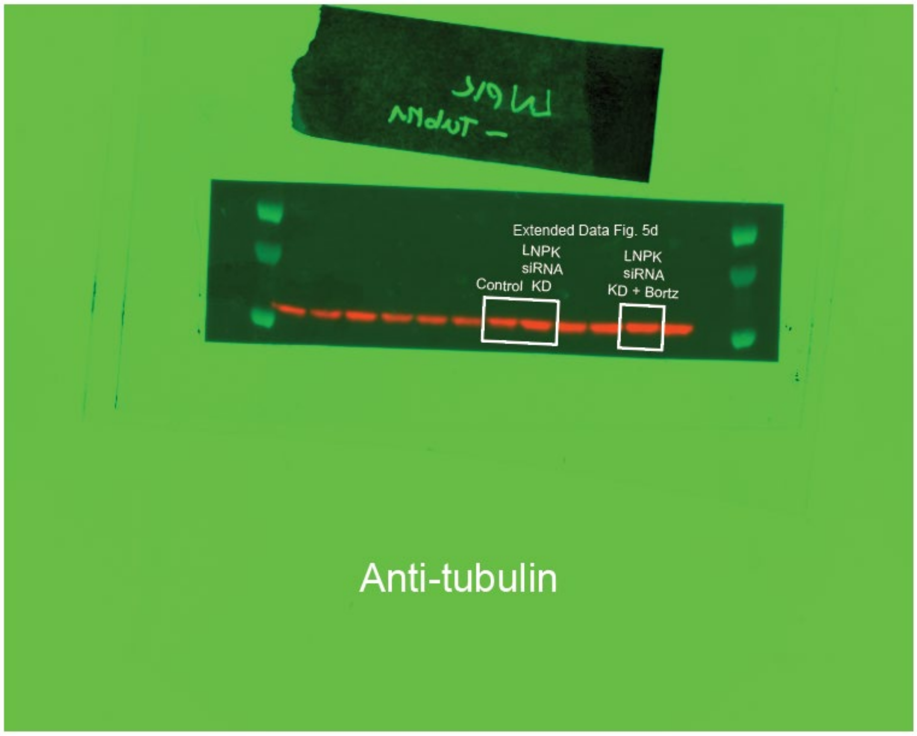

Anti-tubulin

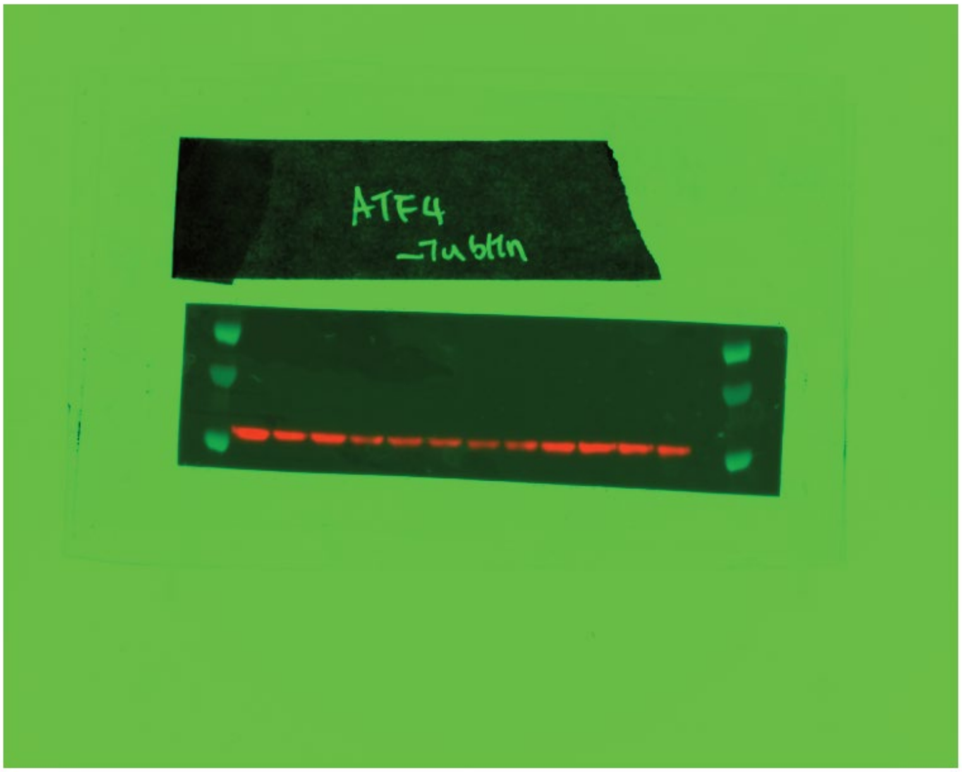
ATF4
-Tubulin

IV. Motion-based analysis and its assumptions

To formalize the MSD-based classification, the probability distribution of effective 2D diffusion coefficient, $p(D_{eff})dD_{eff}$ where $D_{eff} = \langle r^2 \rangle/(4*i\Delta t)$, $\langle r^2 \rangle$ = mean squared displacement, and $i\Delta t$ = time lag, is described in [1] and with Equation 1 (Eq.1)

$$p(D_{eff}) = \frac{1}{(N-1)!}\left(\frac{N}{D_0}\right)^N (D_{eff})^{N-1} e^{-\frac{N D_{eff}}{D0}} \quad \text{(Eq.1)}$$

where $N = N_{total}/ i\Delta t$, N_{total} = length of trajectory, D_0 = true diffusion coefficient, D_{eff} = effective diffusion coefficient for an individual trajectory based on its squared displacement. As N increases, the distribution narrows. Assuming translation is highly processive and that state switching (translating or not translating) does not occur within the imaging window, translating and non-translating mRNAs can be approximated as two independent populations with distinct D_0. To explore the separation required for averaging, we performed Monte Carlo simulations of 5,000 trajectories with diffusion coefficients of 0.007 μm²/s (SUNTAG(+)) and 0.033 μm²/s (puromycin-treated). Histograms of averaged steps revealed that false positives below the $MSD_{T=1s}$ cut-off of 0.055 μm² are possible, particularly for short trajectories (**Supplementary Fig.1**). Because MS2-tagged secretome mRNAs often yield trajectories of 7–8 steps, while classification requires >9, the method likely overestimates the translating fraction and undercounts short, non-translating trajectories. Puromycin treatment substantially reduced the apparent translating pool, confirming the method's sensitivity to changes in translation state, but results should be treated as relative rather than absolute measures.

In this study, single-molecule tracking of MS2- or SUNTAG-labeled mRNAs was analyzed using mean squared displacement (MSD) thresholds to classify translational states. Puncta with confined motion below the MSD cut-off were interpreted as translating, whereas more mobile puncta were considered non-translating. To ensure reliability, only trajectories longer than ten steps were included, which biases the analysis toward longer-lived, confined puncta and underrepresents fast-diffusing, non-translating pools. In addition, nuclear regions were masked to avoid tracking artifacts caused by MCP accumulation in the nucleus. This procedure removes signals near the nuclear periphery and may obscure a subset of ER-associated transcripts (**Supplementary Fig. 2a**). For these reasons, translation fractions derived from MSD-based thresholds should be viewed as conservative estimates rather than absolute measurements.

For ER-bound mRNA analysis, ER membranes were segmented and skeletonized to identify junctions, followed by nearest-neighbor assignment of tracked mRNAs (**Supplementary Fig. 2b**). The accuracy of this approach depends heavily on image resolution and segmentation quality, and can be confounded in regions of high curvature or dense ER tubules. To minimize misassignments, junction localization was validated by manual curation.

Finally, lysosome proximity analysis was performed by simultaneously tracking LAMP1-positive vesicles, mRNAs, and nascent peptide signals (**Supplementary Fig. 2c**). In these experiments, correlation of SUNTAG signal intensity with lysosome distance was used to assess ribosome load, with brightness serving as a proxy for the number of translating ribosomes. While this approach reliably reports reproducible

enrichment of bright SUNTAG(+) puncta near lysosomes, it cannot distinguish between stalled ribosomes and actively elongating ribosomes. Thus, FRAP of SUNTAG spots may be ideal to distinguish stalled vs active elongating ribosomes.

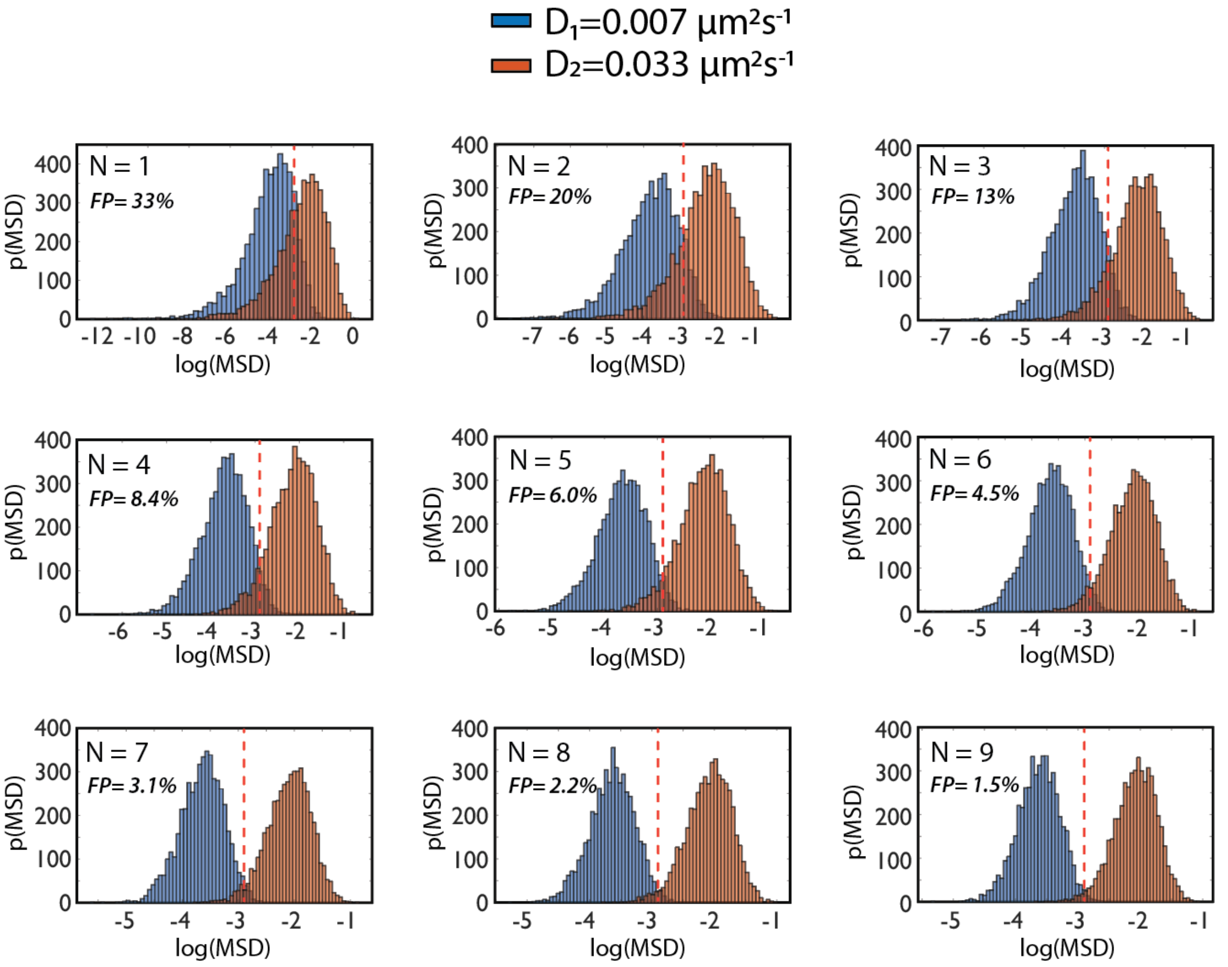

Supplementary Fig. 1. MSD simulations separate translating from nontranslating mRNAs Probability distribution of mean squared displacement (MSD) values for simulated trajectories with diffusion coefficients of 0.007 µm²/s (blue, matching SUNTAG(+) mRNAs) and 0.033 µm²/s (orange, matching puromycin-treated SiT-EGFP mRNAs). Distributions shown for increasing N-step averaging. As N increases, separation between slow and fast populations improves, reducing overlap. False positive (FP) rates calculated relative to the translation cut-off (red dotted line at MSD=0.055 µm²). FP rates decrease as trajectory averaging length increases, confirming the robustness of the MSD threshold used to distinguish translating vs non-translating mRNAs.

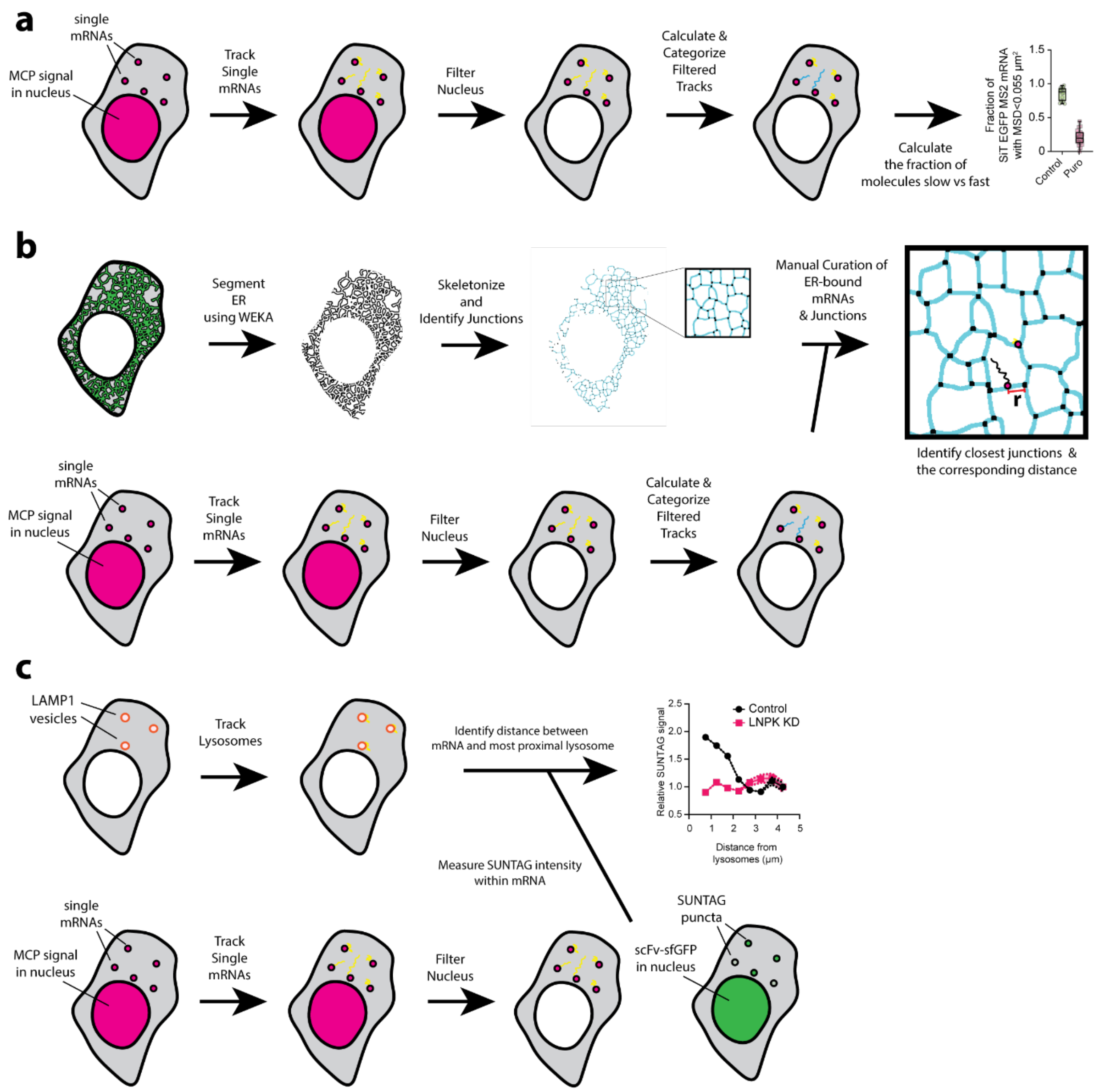

Supplementary Fig. 2. a, Schematic illustrating the approach for tracking, filtering, and categorizing mRNA movements. Individual mRNAs were tracked over time, and signals from the nucleus were masked due to high MCP accumulation resulting from the nuclear localization signal (NLS). Filtered mRNA tracks were categorized based on mean squared displacement, calculated over a 10-second interval with a 1-second lag time. **b,** Schematic depicting the method for monitoring ER-bound mRNAs. The ER structure was segmented using WEKA or ilastik. The resulting mask was skeletonized, and junctions were identified using a custom-built algorithm for detecting branching points. Concurrently, mRNAs were tracked as described in Extended Data Fig. 6a, and their distances from the nearest ER junctions were determined by nearest neighbor algorithm. The accuracy of junction identification and the actual ER association of mRNAs were further validated through manual curation. **c,** Schematic illustrating the

quantification of distances between cytERM-SUNTAG-MS2 mRNAs and lysosomes. LAMP1 vesicles were tracked using TrackMate, while mRNA and scFv-sfGFP signals were tracked as described in Extended Data Fig. 6. SUNTAG-associated mRNA signals were identified, and distances between lysosomes and corresponding mRNAs were calculated.

Supplementary Table 1. List of Plasmids for this study

Name	Source
pUBC-SiT-EGFP-MS2	This study
pUBC-Calreticulin-mEmerald-MS2	This study
pUBC-CD4-EGFP-MS2	This study
pUBC-Halo-Actin-MS2	[2]
pCMV-SNAPf-Sec61b	This study
pCMV-mApple-Sec61b	[3]
pCMV-LAMP1-Halo-iLID	This study
pCMV-sspB-mCherry-Sec61	This study
pCMV-L10A-Halo	This study
pPGK-Lamp1-mApple	[4]
pPGK-Lamp1-mScarlet-i	This study
pPB-cytERM-SUNTAG-MS2	This study
pPB-CrPV-IRES-cytERM-SUNTAG-MS2	This study
pUBC-MCP-Halo	[2]
pUBC-MCP-GFP	[2]

Supplementary Table 2. siRNA and qPCR primers used for this study

**siRNA sequences used in this study**	
CLIMP63 siRNA SMARTpool Sequences	
siRNA CKAP4, D-012755-01	CCAAAUCCAUCAACGACAA
siRNA CKAP4, D-012755-02	CCAAGGGUUUACUAGAUGA
siRNA CKAP4, D-012755-03	AGACCCAGCUGGUGCUCUA
siRNA CKAP4, D-012755-04	GAGCGUGCAUACUGCGUUU
Lunapark siRNA SMARTpool Sequences	
siRNA KIAA1715, D-023148-01	GGUAUGCACUUAUAUGUCA
siRNA KIAA1715, D-023148-02	AUUAUGGGUUGGAAGAUUA
siRNA KIAA1715, D-023148-03	ACGAUGUUCUUGAUGAUAA
siRNA KIAA1715, D-023148-04	CGGCUAAAUUAAUUCUUGA
qPCR Primer sets	
Lunapark (LNPK)	
Forward Set 1	CTC CTC CAC AAG TTC CAG TAT
Reverse Set 1	GAT GAT AGG GCT GGA GTA ACA G
Forward Set 2	ACT GTT ACT CCA GCC CTA TCA
Reverse Set 2	CTA TCC AAA GCA CCT CGT TCT C
Forward Set 3	GCA AGA CCT GGA CAA GAG ATT
Reverse Set 3	TTG GTG GTC CAG GAG ATA CT
CLIMP63 (CKAP4)	
Forward Set 1	GGA ATC AGC CAA GGG TTT ACT A
Reverse Set 1	CCT GCA CAC GCA ATT CAT TTA
Forward Set 2	GGA AGC TGT GAA GGA GAT ACA G
Reverse Set 2	ACC TCG GTG TAG ATG TCA GA
Forward Set 3	TGG TTG CAT ACT CGG TCA AA
Reverse Set 3	CCA GAT CAT TCC TCA GGT CAT C
β-Actin (ActB)	
Forward Set 1	CAC CAT TGG CAA TGA GCG GTT C
Reverse Set 1	AGG TCT TTG CGG ATG TCC ACG T
Unspliced XBP1 for human	
Forward Set 1	CAG ACT ACG TGC ACC TCT GC
Reverse Set 1	CTG GGT CCA AGT TGT CCA GAA T
Spliced XBP1 for human	
Forward Set 1	GCT GAG TCC GCA GCA GGT
Reverse Set 1	CTG GGT CCA AGT TGT CCA GAA T
Total XBP1 for human	
Forward Set 1	TGA AAA ACA GAG TAG CAG CTC AGA
Reverse Set 1	CCC AAG CGC TGT CTT AAC TC

Supplementary Table 3. Single guide RNAs for generating Lunapark KO U-2 OS cells

**Lunapark KO Generation**	
gRNA sequence targeting Human Lunapark	
sgRNA-LNPK-1	AGCAAAAAAUGGGAGUGUCA
sgRNA-LNPK-1	UGUAAACAGAUAGAGAACUG
sgRNA-LNPK-1	UCAAGCAUUGGAAGAAUUUA
Amplification and sequencing primers	
KIAA1715 Forward-1	CCTCAGGTATATTGGATTACAAATGAAGG
KIAA1715 Reverse-1	TGCCGATACCAATTTCATTCCC
KIAA1715 Seq-1	CCTCAGGTATATTGGATTACAAATGAAGG
KIAA1715 Reverse-2	TCATTCCCTGAATCTTTGCCA

Supplementary Video 1

SiT-EGFP MS2 mRNA (red) on ER marked by SNAPf-Sec61β (cyan) in U-2 OS cells. Time is in second. The scale bar represents 5 μm.

Supplementary Video 2

Categorized SiT-EGFP MS2 mRNA on ER marked by SNAPf-Sec61β (yellow) in U-2 OS cells. Magenta represents SiT-EGFP MS2 mRNAs with $MSD_{\tau=1sec} > 0.055$ μm2. Green represents SiT-EGFP MS2 mRNAs with $MSD_{\tau=1sec} < 0.055$ μm2. Time is in second. The scale bar represents 5 μm.

Supplementary Video 3

Categorized SiT-EGFP MS2 mRNA on ER marked by SNAPf-Sec61β (yellow) in U-2 OS cells under puromycin treatment. Magenta represents SiT-EGFP MS2 mRNAs with $MSD_{\tau=1sec} > 0.055$ μm2. Green represents SiT-EGFP MS2 mRNAs with $MSD_{\tau=1sec} < 0.055$ μm2. Time is in second. The scale bar represents 5 μm.

Supplementary Video 4

SUNTAG(+) cytERM-SUNTAG MS2 mRNA movie. Anti-GCN4 scFv-sfGFP (SUNTAG, Green) and MCP-Halo (RNA, Magenta). Time is in second. The scale bar represents 1 μm.

Supplementary Video 5

SUNTAG(-) cytERM-SUNTAG MS2 mRNA movie. Anti-GCN4 scFv-sfGFP (SUNTAG, Green) and MCP-Halo (RNA, Magenta). Time is in second. The scale bar represents 1 μm.

Supplementary Video 6

cytERM-SUNTAG MS2 mRNA movie under puromycin treatment. Anti-GCN4 scFv-sfGFP (SUNTAG, Green) and MCP-Halo (RNA, Magenta). Time is in second. The scale bar represents 1 μm.

Supplementary Video 7

SUNTAG-Sec61β MS2 mRNA movie. Anti-GCN4 scFv-sfGFP (SUNTAG, Green) and MCP-Halo (RNA, Magenta). Time is in second. The scale bar represents 1 μm.

Supplementary Video 8

iLID-Halo-LAMP1 (magenta) and sspB-mCherry-Sec61β (green) localization in control U2-OS cells. Time is in second. The scale bar represents 1 μm. The recruitment by exposing 488 nm was performed at t = 0 sec.

Supplementary Video 9

iLID-Halo-LAMP1 (magenta) and sspB-mCherry-Sec61β (green) localization in Lunapark KO U2-OS cells. Time is in second. The scale bar represents 1 μm. The recruitment by exposing 488 nm was performed at t = 0 sec

References

1. Vrljic, M., Nishimura, S. Y., Brasselet, S., Moerner, W. E. & McConnell, H. M. Translational Diffusion of Individual Class II MHC Membrane Proteinsin Cells. *Biophysical Journal* **83**, 2681-2692 (2002). https://doi.org/10.1016/s0006-3495(02)75277-6
2. Yoon, Y. J. *et al.* Glutamate-induced RNA localization and translation in neurons. *Proceedings of the National Academy of Sciences* **113**, E6877-E6886 (2016). https://doi.org/10.1073/pnas.1614267113
3. Nixon-Abell, J. *et al.* Increased spatiotemporal resolution reveals highly dynamic dense tubular matrices in the peripheral ER. *Science* **354** (2016). https://doi.org/10.1126/science.aaf3928
4. Liao, Y.-C. *et al.* RNA Granules Hitchhike on Lysosomes for Long-Distance Transport, Using Annexin A11 as a Molecular Tether. *Cell* **179**, 147-164.e120 (2019). https://doi.org/10.1016/j.cell.2019.08.050
