# Supplementary material for: Secretome translation shaped by lysosomes and lunapark-marked ER junctions
Source: Nature. 2025 Nov 5;649(8095):227–36. doi: 10.1038/s41586-025-09718-0 (PMC12727531; doi:10.1038/s41586-025-09718-0)
Supplement: Supplementary file 2 — Reporting Summary [file 41586_2025_9718_MOESM2_ESM.pdf]

nature portfolio

Corresponding author(s): Jennifer Lippincott-Schwartz

Last updated by author(s): 2025-11-03

Reporting Summary

Nature Portfolio wishes to improve the reproducibility of the work that we publish. This form provides structure for consistency and transparency in reporting. For further information on Nature Portfolio policies, see our Editorial Policies and the Editorial Policy Checklist.

Statistics

For all statistical analyses, confirm that the following items are present in the figure legend, table legend, main text, or Methods section.

n/a	Confirmed	
☐	☒	The exact sample size (*n*) for each experimental group/condition, given as a discrete number and unit of measurement
☐	☒	A statement on whether measurements were taken from distinct samples or whether the same sample was measured repeatedly
☐	☒	The statistical test(s) used AND whether they are one- or two-sided<br>*Only common tests should be described solely by name; describe more complex techniques in the Methods section.*
☒	☐	A description of all covariates tested
☐	☒	A description of any assumptions or corrections, such as tests of normality and adjustment for multiple comparisons
☐	☒	A full description of the statistical parameters including central tendency (e.g. means) or other basic estimates (e.g. regression coefficient) AND variation (e.g. standard deviation) or associated estimates of uncertainty (e.g. confidence intervals)
☐	☒	For null hypothesis testing, the test statistic (e.g. *F*, *t*, *r*) with confidence intervals, effect sizes, degrees of freedom and *P* value noted<br>*Give P values as exact values whenever suitable.*
☒	☐	For Bayesian analysis, information on the choice of priors and Markov chain Monte Carlo settings
☒	☐	For hierarchical and complex designs, identification of the appropriate level for tests and full reporting of outcomes
☒	☐	Estimates of effect sizes (e.g. Cohen's *d*, Pearson's *r*), indicating how they were calculated

Our web collection on statistics for biologists contains articles on many of the points above.

Software and code

Policy information about availability of computer code

Data collection	Imaging data were acquired on customized Nikon TiE microscopes configured for spinning-disk and HILO illumination with Andor EM-CCD cameras, using Nikon Elements AR 6.0 for spinning-disk imaging and ZEN Black 2.3/Zen Blue for confocal imaging. Western blot signals were captured on a Bio-Rad ChemiDoc system. RNA sequencing was performed on an Illumina NextSeq 2000, and mass spectrometry was carried out using a Thermo Orbitrap Ascend Tribrid mass spectrometer coupled to a Vanquish Neo LC system.
Data analysis	MATLAB (custom scripts for MSD analysis, and Monte Carlo simulations), Imaris, Illastik, Fiji, TrackMate (Fiji), Imaris, CellProfiler, STAR (v2.7.5c) for RNA-seq alignment, DESeq2 in MATLAB for transcriptome analysis, and DIA-NN (v1.9.1) for mass spectrometry

For manuscripts utilizing custom algorithms or software that are central to the research but not yet described in published literature, software must be made available to editors and reviewers. We strongly encourage code deposition in a community repository (e.g. GitHub). See the Nature Portfolio guidelines for submitting code & software for further information.

April 2023

Data

Policy information about availability of data

All manuscripts must include a data availability statement. This statement should provide the following information, where applicable:

- Accession codes, unique identifiers, or web links for publicly available datasets
- A description of any restrictions on data availability
- For clinical datasets or third party data, please ensure that the statement adheres to our policy

Source data underlying all graphs are provided with the paper in FigShare (DOI:10.25378/janelia.30153850). Datasets of single-molecule imaging datasets (HILO and spinning disk confocal movies of MS2- and SUNTAG-labeled secretome mRNAs, ribosome tracking, and lysosome recruitment assays), processed particle-tracking files, and quantified fluorescence recovery after photobleaching (FRAP) measurements have been deposited in FigShare (DOI:10.25378/janelia.30153850). Uncropped gel and blot images corresponding to Figs. 5e and Extended Data Fig. 4 are provided in Supplementary Information. All listed plasmids are deposited to Addgene.

Research involving human participants, their data, or biological material

Policy information about studies with human participants or human data. See also policy information about sex, gender (identity/presentation), and sexual orientation and race, ethnicity and racism.

Reporting on sex and gender	N/A
Reporting on race, ethnicity, or other socially relevant groupings	N/A
Population characteristics	N/A
Recruitment	N/A
Ethics oversight	N/A

Note that full information on the approval of the study protocol must also be provided in the manuscript.

Field-specific reporting

Please select the one below that is the best fit for your research. If you are not sure, read the appropriate sections before making your selection.

☒ Life sciences ☐ Behavioural & social sciences ☐ Ecological, evolutionary & environmental sciences

For a reference copy of the document with all sections, see nature.com/documents/nr-reporting-summary-flat.pdf

Life sciences study design

All studies must disclose on these points even when the disclosure is negative.

Sample size	Sample sizes were chosen based on established practice for single-molecule imaging and translation studies, and were sufficient to detect significant differences. Exact n values (cells, trajectories, replicates) are reported in each figure legend. For example, thousands of individual mRNA trajectories across 13–40 cells were analyzed per condition, and western blots were repeated with three independent lysates.
Data exclusions	Trajectories shorter than 10 steps were excluded from MSD-based classification, as these could not be reliably analyzed. Nuclear signals were excluded due to MCP accumulation, and poor-quality images were omitted prior to analysis.
Replication	All key findings were reproduced in at least two independent biological replicates. RNA-seq and proteomics experiments included biological replicates.
Randomization	We did not randomize any aspects of this study.
Blinding	Data analysis was performed using automated pipelines (MATLAB, TrackMate, Imaris) without prior knowledge of sample identity. Manual validation of ER junction assignments and PLA puncta was performed without knowledge of condition where feasible.

Reporting for specific materials, systems and methods

We require information from authors about some types of materials, experimental systems and methods used in many studies. Here, indicate whether each material, system or method listed is relevant to your study. If you are not sure if a list item applies to your research, read the appropriate section before selecting a response.

Materials & experimental systems

n/a	Involved in the study
☐	☒ Antibodies
☐	☒ Eukaryotic cell lines
☒	☐ Palaeontology and archaeology
☒	☐ Animals and other organisms
☒	☐ Clinical data
☒	☐ Dual use research of concern
☒	☐ Plants

Methods

n/a	Involved in the study
☒	☐ ChIP-seq
☒	☐ Flow cytometry
☒	☐ MRI-based neuroimaging

Antibodies

Antibodies used

Rabbit LNPK antibody (Sigma, HPA014205-25), Mouse EEA1 antibody (BD Biosciences, 610456), Mouse LAMP1 antibody (Abcam, ab25630), Mouse TOMM20 antibody (ProteinTech, 66777-1-Ig).Rabbit anti-ATF-4 antibody (Cell Signaling, 11815S), Rabbit anti-LNPK antibody (Sigma, HPA014205-25), Mouse anti-Tubulin antibody (Millipore, 05-829), Rabbit anti-eIF2S1 (phosphor S51) antibody (Abcam, ab32157), and Rabbit anti-eIF2S1 antibody (Atlas Antibodies, HPA064885). Secondary antibodies used here are: Goat anti-mouse Ig H&L-HRP (Abcam, ab205719), Goat anti-rabbit Ig H&L-HRP (Abcam, ab205718), Rabbit REEP5 antibody (ProteinTech, 14643-1-AP).

Validation

Refer to manufacturer's website

Eukaryotic cell lines

Policy information about cell lines and Sex and Gender in Research

Cell line source(s)

All experiments in the paper are performed using U-2 OS, COS7, HEK293T, HeLa, and HT1080 cells from ATCC, experiments are performed within 40 passages of the initial shock provided.

Authentication

We have not performed any authentication of the lines. There were no obvious differences in any property examined.

Mycoplasma contamination

Cells were all free of mycoplasma at the time of experimentation, and are tested routinely during passaging.

Commonly misidentified lines
(See ICLAC register)

No commonly misidentified cell lines were used in this study.

Plants

Seed stocks

N/A

Novel plant genotypes

N/A

Authentication

N/A
